# Imaging Fos-Jun Transcription Factor Mobility and Interaction in Live Cells by Single Plane Illumination-Fluorescence Cross Correlation Spectroscopy

**DOI:** 10.1371/journal.pone.0123070

**Published:** 2015-04-14

**Authors:** Agata Pernuš, Jörg Langowski

**Affiliations:** Division Biophysics of Macromolecules, DKFZ, Heidelberg, Germany; Beijing Forestry University, CHINA

## Abstract

We collected mobility and interaction maps of c-Fos-eGFP and c-Jun-mRFP1 transcription factors within living cell nuclei. c-Fos dimerizes with c-Jun to form the transcription activator protein-1 (AP-1) which binds to the specific recognition site. To monitor this process, we used fluorescence cross-correlation spectroscopy on a single plane illumination microscope (SPIM-FCCS), which provides diffusion coefficient and protein-protein interaction data in the whole image plane simultaneously, instead of just one point on conventional confocal FCS. We find a strong correlation between diffusional mobility and interaction: regions of strong interaction show slow mobility. Controls containing either an eGFP-mRFP dimer, separately expressing eGFP and mRPF, or c-Fos-eGFP and c-Jun-mRFP1 mutants lacking dimerization and DNA-binding domains, showed no such correlation. These results extend our earlier findings from confocal FCCS to include spatial information.

## Introduction

Molecular dynamics inside cells can be investigated by different fluorescence fluctuations microscopy methods [1]. Fluorescence correlation spectroscopy (FCS) [2, 3] is a well-known tool to study molecular motions *in vitro* and *in vivo* [4–6]. This technique is typically implemented on a confocal microscope, which enables detection of fluorescently labeled molecules with single-molecule sensitivity using avalanche photodiode detectors [7, 8]. However, for understanding cellular mechanisms it is important to characterize not only single molecular species at isolated points, but to study them spatially resolved across the entire cell in the context of their interaction partners to understand their function [9]. Two-color fluorescence cross-correlation spectroscopy (FCCS) is a well-established tool for detecting biomolecular interactions [10–12]. With this technique, two molecules are labeled with different fluorophores to distinguish them spectrally. Cross-correlating the fluorescence fluctuations in the detection channels will then reveal their interaction. Several studies utilizing FCCS have concentrated on the interaction and localization of membrane-bound proteins [13, 14] and the dynamics of the nuclear receptors RAR [15] and RXR [16].

Current FCCS allows routine measurements of biomolecular interactions in living cells [17–21] on a confocal microscope, however its drawback is that measurements can be performed only at one or at most a few spots at a time. Although FCCS can provide information on the mobility of proteins and their interaction at selected spots [7], data collection is tedious and slow, and motion of cells hampers data collection. A technique that allows imaging in a thin plane and spatially resolved FCCS by analysis of fast image series is selective plane illumination microscopy (SPIM). SPIM [22, 23] illuminates the fluorescently labeled sample by a thin light sheet formed by a laser beam focused through cylindrical optics and simultaneously images all points in the two-dimensional field of view with a fast camera. As it reduces the exposure of the cells to light, much longer observation times are possible.

Camera-based SPIM-FCS was introduced recently, characterizing fluorescent beads moving in the bloodstream of living zebrafish embryos [24], or the mobility of the heterochromatin protein HP1α in the cell nucleus [25]. To extend SPIM-FCS to SPIM-FCCS, the sample is excited with two overlapping light sheets of different wavelengths and the two fluorescence channels are optically split to two half planes of the same sensor. This allows simultaneous analysis of fluorescence fluctuations at every point of the image. The time resolution of SPIM-FCCS is determined by the speed of the recording camera. High speed electron-multiplying charge coupled device (EMCCD) cameras have high detection efficiency with frame rates in the 1000 s^-1^ range [24, 26–30]. In comparison to other detectors [31], EMCCD cameras proved to be the best choice in the sense of acquisition speed and photosensitivity. Very recently, a SPIM-FCCS system has been described in detail, its feasibility demonstrated [32, 33] and some applications of imaging FCS have been described [34].

The regulation of DNA transcription into RNA and its translation into proteins is one of the most important processes determining cell behavior. Transcription factors (TFs) regulate DNA transcription into RNA, thereby controlling protein expression and cellular processes. General TFs like c-Fos and c-Jun participate in the regulation of processes such as proliferation, differentiation, apoptosis and oncogenesis [35–38]. Their heterodimer formation has been shown *in vitro* [39–42].

Here we use a new technique, FCCS in single plane illumination (SPIM-FCCS) [32, 33], to study the interactions of the AP-1 transcription factors c-Fos and c-Jun in HeLa cells. Earlier FCCS measurements on c-Fos-eGFP / c-Jun-mRFP using single-point measurements on a confocal setup demonstrated their interaction in living cell nuclei [43, 44] and indicated that dimerization is a prerequisite to DNA binding. This finding was in partial agreement with an earlier stopped-flow kinetic study where the association of Fos and Jun was detected by fluorescence resonance energy transfer (FRET) [45] in the absence and presence of DNA, suggesting that the protein monomers prefer to bind to DNA separately and then dimerize.

The aim of this study was to obtain detailed knowledge about the localization, dynamics, interaction and DNA binding of the c-Fos and c-Jun transcription factors (TFs). We characterized spatially resolved interactions and mobility of these TFs inside the cell nucleus by *in vivo* SPIM-FCCS. We show that the dimerization of the TFs leads to a massive slowing down of their motion, indicating that dimerization and binding to genomic DNA are closely connected.

## Materials and Methods

### Plasmid construction

We used expression vectors pSV-c-Fos-eGFP and pSV-c-Jun-mRFP1 constructed by N. Baudendistel using a multi-step cloning strategy described in detail in [43]. Briefly, a pSVEYFP vector originating from pECFP-1 (BD Biosciences, Palo Alto, USA) was used as a starting vector to construct the expression vectors. The SV40 promoter region was inserted in the HindIII restriction site to drive overexpression of the protein constructs. The pSV-c-Fos-eGFP and pSV-c-Jun-mRFP1 consist of full-length human Fos fused to eGFP with a linker sequence RDPPVAT and a Jun fused to mRFP1 with the linker sequence RDPPV cloned to create the protein Jun-mRFP1 [43, 44]. Throughout the text, the resulting fusion proteins are termed as c-Fos-eGFP and c-Jun-mRFP1.

The control vectors pSV-eGFP-mRFP1, a fusion protein of the two dyes separated by a 7-AA linker, and pIRES2-eGFP-mRFP1, expressing the dyes separately, as well as the negative controls c-FosΔΔ-eGFP and c-JunΔΔ-mRFP1 that are lacking the dimerization and DNA-binding domains were also constructed by N. Baudendistel [43].

### Cell culture and transfection

Adherent HeLa cells (provided by F. Rösl, DKFZ, Heidelberg, Germany) were grown at 37°C in a 5% CO_2_ humidified atmosphere in a phenol-free DMEM growth medium (Invitrogen Life Technologies, Carlsbad, USA) with added 10% fetal calf serum and 1% Glutamine. The mammalian expression vectors were transfected with FuGENE HD (Promega GmbH, Mannheim, Germany) as proposed by the manufacturer (Table 1). For the SPIM-FCCS measurements, HeLa cells were plated and transfected on small glass pieces in a 35 mm petri dish 24–48 h before the measurements. For confocal FCCS experiments, cells were seeded on 32 mm cover slides in a 60 mm petri dish [7]. The transfection procedure [43] was the same as for the SPIM measurements.

**Table 1:** Transfection protocols of HeLa cells used for the measurements (for the standard 35 mm petri dish).

Plasmid	Amount of Plasmid	Transfection
c-Fos-eGFP and c-Jun-mRFP1	1–1.5 μg	45 μl DMEM medium4 μl FuGENE HD
c-FosΔΔ-eGFP and c-JunΔΔ-mRFP1	1–1.5 μg	“
eGFP-mRFP1 fusion protein	100 ng	“
eGFP, mRFP1 monomers (IRES)	140 ng	“

### Confocal FCCS

The quantitative results obtained by SPIM-FCCS were verified with the established confocal FCCS method. Control FCCS measurements were conducted on an in-house constructed setup built around an inverted Olympus IX-70 microscope (Olympus, Hamburg, Germany) with a 60x / NA = 1.2 water immersion objective [46, 47]. An integrated galvanometer scanner allowed imaging and selecting the FCCS focus spot. The sample was excited with an argon-krypton laser (CVI Melles Griot, Bensheim, Germany) with 488 nm and 568 nm wavelengths at intensities of ~2 kW/cm^2^. Fluorescence was separated from the excitation light by a dichroic mirror and detected in two color channels with single photon counting avalanche photodiodes (SPCM-AQR-13, Perkin-Elmer, Wellesley, USA). Real-time computation of photon correlation functions was performed on an ALV-5000 multi-tau correlator card (ALV Laser GmbH, Langen, Germany). The card allows computation of cross-correlation as well as auto-correlations functions. The system was calibrated with Alexa 488 [48] and Alexa 594 [49] as described in [43]. We acquired confocal images and performed auto- and cross-correlation measurements at 4–5 selected points of 20 cells of each of the constructs listed in Table 1. Data acquisition time at a selected point was 60 s, consisting of 6 rounds of 10 s. Auto- and cross-correlation functions obtained by confocal microscope measurements were fitted to a model function assuming two diffusing fluorescent components (D_fast_, D_slow_) and a triplet correction [43], using the Marquardt-Levenberg algorithm. The correlation functions were normalized to the smallest of the two auto-correlation amplitudes. The amount of binding was defined as the relative cross-correlation (CCF) amplitude (*q*) and was calculated by dividing the cross-correlation function by the smaller of the two auto-correlation functions:

$$q=\frac{g_{gr}(\tau )}{min[g_{gg}(\tau ),g_{rr}(\tau )]}$$ (1)

where τ is the minimum lag time. All data evaluation was performed with *QuickFit 3*.*0* [50].

### Single plane illumination microscopy with dual color excitation and detection

**Fig 1:**
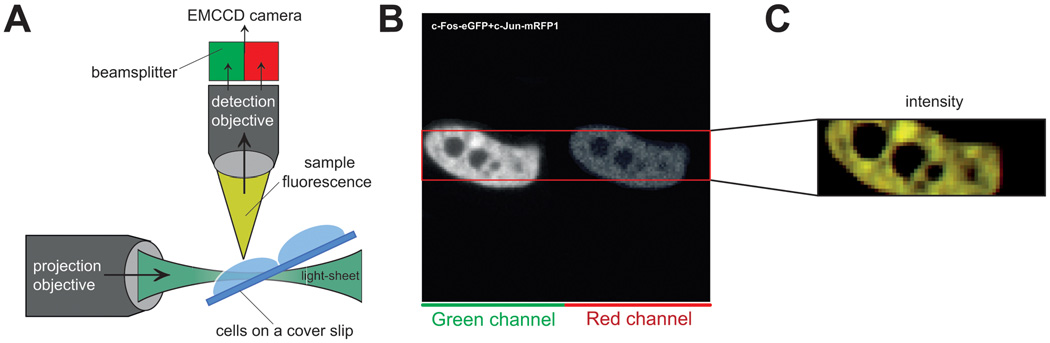
(A) SPIM-FCCS principle. (B) example of a SPIM-FCCS full-frame fluorescence image. The marked area indicates the measurement region (128x20). (C) total intensity image obtained by combining the images in the green and red channels.

For SPIM-FCS/FCCS measurements, we used a home-built dual color excitation and detection single plane illumination microscopy system (SPIM) based on the design in [22, 23]. The detailed description is given in Krieger et al. [32, 33]. In brief, a blue 491 nm (Cobolt Calypso, Sweden, 25 mW) and a green 561 nm (Cobolt Jive, Sweden, 25 mW) laser beam with two distinct beam expanders were combined by a dichroic mirror into a dual color excitation beam. The combined beam was then relayed by a telescope into a cylindrical lens followed by a projection objective, which formed an approximately 1.3 μm thick (1/e^2^-halfwidth) light sheet illuminating the cells. The glass piece with the adherent HeLa cells was clamped and mounted from above at an angle slightly below 45° to the light sheet into the sample chamber filled with Hanks’ solution (Fig 1A).

The sample fluorescence was collected perpendicular to the illumination by a water-dipping objective (Nikon CFI Apo-W NIR 60x/NA 1.0) and corresponding tube lens. Fluorescence was split with an emission splitting system (DualView DV2, Photometrics, Tucson, USA) into two spatially identical and spectrally distinct images. By maximizing the image cross-correlation between the red and green color channels the system was adjusted for best overlap (see below). The two images were simultaneously projected side-by-side onto a 128x128 pixel EMCCD camera (iXon X3 860, Andor, Belfast; quantum efficiency ≈ 95%). The field of view was around 50 μm x 50 μm, which was sufficient to image a HeLa cell with a typical diameter of 20–30 μm, while the pixel size was 400x400 nm². The intensity fluctuations of the two fluorophores at the same spot of a sample were thus detected simultaneously at corresponding pixels in the detector half planes. We measured at laser intensities between 60–100 W/cm^2^ in each light sheet, which is a factor of 20 lower compared to the power density required by confocal FCCS. If we compare the total energy deposited in the cell either by acquiring a SPIM image over time *t*, or FCS data during the same time *t* per spot, the ratio of the total laser energies hitting the cell during the acquisition would be in first approximation equal to the ratio of the light sheet thickness to the total cell thickness in the z direction (5 μm), multiplied by the ratio of the power densities. Thus, the total laser power load in SPIM is about 100 times less than in an equivalent confocal scan. During the (shorter) acquisition, however, the total laser power in the SPIM is higher, making photo-bleaching more prominent and a bleaching correction necessary.

On each day of measurements, the SPIM was calibrated following the procedure described in Krieger et al. [32, 33]. Images in the two color channels were aligned by maximizing the cross-correlation coefficient between the two images of a calibration grid to about 0.98. The lateral point spread functions (PSF) (determined by SPIM-FCS from the diffusion time of 100 nm TetraSpec beads in water) were w_g_ = (610±50) nm and w_r_ = (620±50) nm, while the longitudinal PSFs (determined from the z-scan of 100 nm TetraSpec beads in gel) were z_g_ = (1100±100) nm and z_r_ = (1200±100) nm. The SPIM setup was controlled by hardware control plugins installed in our in-house developed software *Quickfit 3*.*0*, which also served for data analysis [50].

Fig 1C shows a typical example of a SPIM fluorescence HeLa cell image obtained by combining the green and red channel images with c-Fos-eGFP in the left and c-Jun-mRFP1 in the right half of the detector (Fig 1B). For each cell we acquired an image series of 50.000 frames with a frame rate of approximately 1000 fps. Additionally, by turning off the lasers, we acquired 5000 background frames with the same frame rate. The cells were masked with an intensity threshold to exclude pixels outside the cell or in very dark regions like the nucleolus. Before analysis, the background images were averaged and subtracted from the data images. The cross-talk κ_gr_ measured with c-Fos-eGFP only was between 5 and 8%. Cross-talk correction was incorporated in a model fit. Photo-bleaching was corrected independently for every pixel by normalizing the intensity with an exponential function fitted to the pixel’s intensity time trace as described in [51]:

$$f(t)=A\cdot exp[-\frac{t+f_{2}t^{2}+f_{3}t^{3}}{\tau_{B}}]$$ (2)

Two examples of original and photo-bleaching corrected intensities are shown in supplementary information (S10 Fig).

To determine the diffusion coefficients, we used theoretical models for SPIM-FCS in the case of a binding reaction *A + B* ⇌ *AB*. For the evaluation of the auto-correlation and cross-correlation data from each pixel, a global fit model has been chosen, fitting each pixel separately. By a global fitting approach, the optimum set of parameters is found, minimizing the least-squares deviations of the fit functions from the measurements. The average over all pixels is first fitted to give initial values for further single pixel fits, which are repeated 3–5 times to assure all pixels have been appropriately fitted. Three distinct species A (green labeled monomer), B (red labeled monomer) and AB (double-labeled dimer formed from A and B) were assumed in the model functions in Eqs (3)–(5) for the interaction [32, 33]:

$$g_{gg}\left(\tau \right)=\frac{1}{\langle c_{A}\rangle +\langle c_{B}\rangle }\;\cdot \;G_{gg}\left(\tau \right)$$ (3)

$$g_{rr}\left(\tau \right)=\frac{\eta_{r}^{2}\cdot \left[\langle c_{B}\rangle +\langle c_{AB}\rangle \right]+\kappa_{gr}^{2}\eta_{g}^{2}\cdot \left[\langle c_{A}\rangle +\langle c_{AB}\rangle \right]+2\kappa_{gr}\eta_{r}\eta_{g}\langle c_{AB}\rangle }{{\left(\kappa_{gr}\eta_{g}\langle c_{A}\rangle +\left(\eta_{r}+\kappa_{gr}\eta_{g}\right)\cdot \langle c_{AB}\rangle +\eta_{r}\langle c_{B}\rangle \right)}^{2}}\cdot G_{rr}\left(\tau \right)$$ (4)

$$g_{gr}\left(\tau \right)=\frac{\eta_{g}\eta_{r}\langle c_{AB}\rangle +\kappa_{gr}\eta_{g}\eta_{r}\langle c_{A}\rangle +\kappa_{gr}\eta_{g}^{2}\cdot \langle c_{AB}\rangle }{\left(\eta_{g}\langle c_{A}\rangle +\eta_{g}\langle c_{AB}\rangle \right)\cdot \left(\kappa_{gr}\eta_{g}\langle c_{A}\rangle +\left(\eta_{r}+\kappa_{gr}\eta_{g}\right)\langle c_{AB}\rangle +\eta_{r}\langle c_{B}\rangle \right)}\;\cdot \;G_{gr}\left(\tau \right)$$ (5)

where *η*_*g*_ and *η*_*r*_ are the molecular brightnesses of fluorophore A in the green channel and fluorophore B in the red channel and can be estimated from the measured and background corrected average fluorescence intensities [32, 33], *κ*_*gr*_ is the cross-talk of the green into the red channel, *G*(*τ*) is the factor describing the non-normalized (cross-)correlation functions of species A, B or AB between the green and the red channel, and the *c*_*A*,_ *c*_*B*_ and *c*_*AB*_ are the concentrations. To reduce the complexity of the model, the concentrations *c*_*A*,_ *c*_*B*_ and *c*_*AB*_ were linked over all three curves during the fit. The relative concentration (*p*_*AB*_), which estimates the amount of binding, was calculated as:

$$p_{AB}=\frac{c_{AB}}{c_{A}+c_{B}+c_{AB}}$$ (6)

In the fitting model described by Eqs (3)–(5), a slow (D_slow_) and a fast (D_fast_) diffusion coefficient and their relative contributions (fractions) describe the correlation curves for the green and the red channels by fast and a slow component. The diffusion coefficients were specific to each detection channel and not to the species. By color coding the fast and slow diffusion coefficients, relative concentration and fraction of the slow diffusion component values at each pixel, we constructed parameter images.

The data from all constructs (wild type, deletion mutants, and controls) were fitted with the same SPIM-FCS 2-component normal diffusion model. For the negative and positive controls, it was assumed that the proteins have the same size and thus the same diffusion coefficients, therefore the three diffusion coefficients were linked together during the fit (*D*_A_ = *D*_B_ = *D*_AB_).

SPIM-FCCS *in vivo* measurements were carried out at room temperature (22°-24°C) on HeLa cells expressing either c-Fos and c-Jun, their deletion mutants, eGFP and mRFP1 monomers (negative control) or eGFP-mRFP1 fusion proteins (positive control). Twenty cells of each construct with low fluorescence intensity, corresponding to low protein concentrations, were selected for analysis. If a cell had moved during the measurement, the data was discarded. Measurements were obtained at 60 x 20 pixels of the EMCCD camera, a sub-region that covered most of each cell.

## Results and Discussion

**Fig 2:**
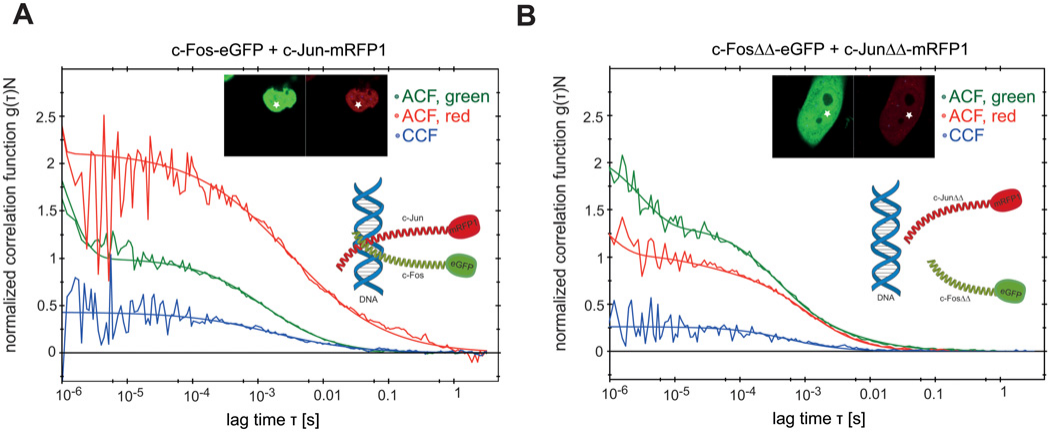
Auto- and cross-correlation functions obtained by confocal *in vivo* FCCS measurements on (A) AP-1 wild-type proteins c-Fos-eGFP and c-Jun-mRFP1 and (B) AP-1 deletion mutants c-FosΔdimΔDNA-eGFP and c-JunΔdimΔDNA-mRFP1. The point of measurement is indicated in cell images.

To confirm the results obtained by Baudendistel et al. [43, 44] and to verify the novel results obtained by the SPIM-FCCS, we have performed control pointwise confocal FCCS measurements on 20 HeLa cells of each construct, expressing either c-Fos and c-Jun, their deletion mutants, eGFP and mRFP1 monomers or eGFP-mRFP1 fusion proteins. Fig 2 shows examples of confocal FCCS measurements of c-Fos-eGFP and c-Jun-mRFP1 (Fig 2A) and its mutants (Fig 2B). The correlation functions were fitted with a global 2-component normal diffusion fit and yielded (mean±SD) diffusion coefficients for c-Fos and c-Jun of (0.25±0.05) μm²/s for slow and (15±3) μm²/s for fast moving molecules. The fast component indicates freely diffusing proteins, whereas the slow component indicates that the AP-1 proteins are immobilized and that this complex might be associated with DNA. Control measurements were performed with eGFP, mRFP1 monomers and the eGFP-mRFP1 fusion protein and respectively served as a reference for minimum and maximum interaction *in vivo*. The relative cross-correlation amplitude *q* obtained for the deletion mutants was (0.18±0.05), which was close to the minimal value (0.15±0.05) obtained for eGFP, mRFP1 monomers. The relative cross-correlation amplitude *q* for the c-Fos-eGFP and c-Jun-mRFP1 was (0.35±0.05), which indicates their dimerization, as the value for the eGFP-mRFP1 fusion protein was (0.45±0.05). The *in vivo* confocal FCCS results that are summarized in Table 2 confirmed the finding of Baudendistel et al. [43, 44] that dimerized transcription factors are probably bound to the DNA.

**Table 2:** Summary of the SPIM-FCCS and confocal results: Diffusion coefficients Dfast and Dslow, relative concentration *p*_*AB*_ (SPIM-FCCS) and relative cross-correlation amplitudes *q* (confocal FCCS).

	SPIM-FCCS			Confocal FCCS		
CELLS	D_fast_ [μm²/s]	D_slow_ [μm²/s]	*p*_*AB*_	D_fast_ [μm²/s]	D_slow_ [μm²/s]	*q*
c-Fos-eGFP,c-Jun-mRFP1	18±1023±6	0.28±0.10.3±0.1	0.21±0.18	15±3	0.25±0.05	0.35±0.05
c-FosΔΔ-eGFP,c-JunΔΔ-mRFP1	22±1023±7	0.38±0.20.4 ± 0.2	0.13±0.07	23±4	0.3±0.05	0.18±0.05
eGFP-mRFP1fusion protein	30±20	0.39±0.2	0.29±0.15	20±3	0.3±0.05	0.45±0.05
eGFP, mRFP1monomer protein	38±15	0.38±0.2	0.08±0.08	30±3	0.3±0.05	0.15±0.5

The SPIM-FCCS and confocal FCCS measurements, each obtained on different days, were averaged over 20 cells.

**Fig 3:**
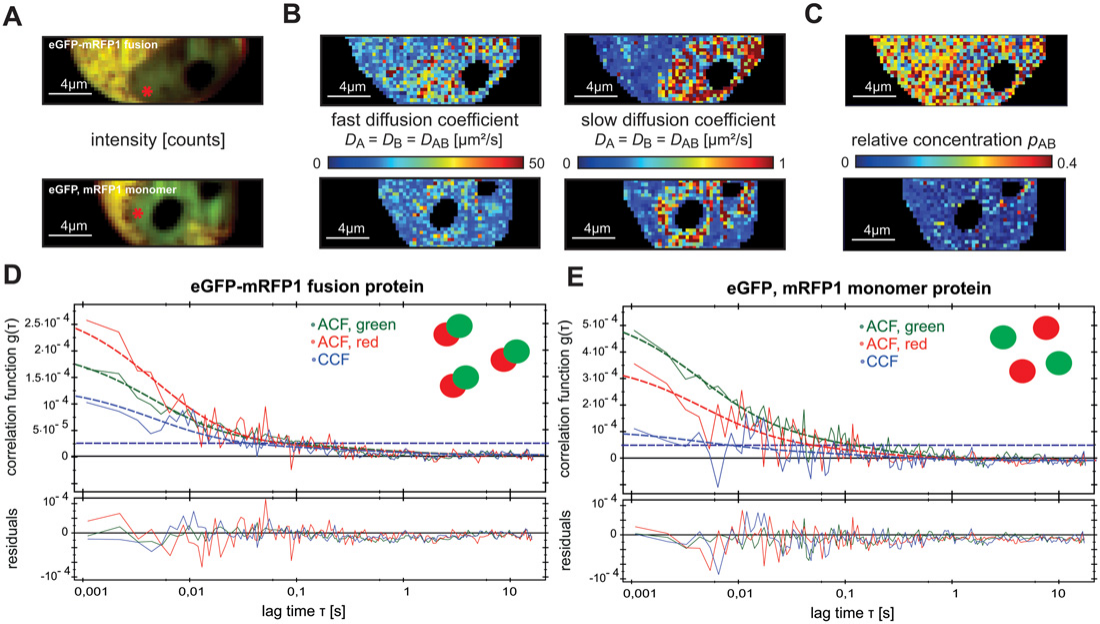
A SPIM-FCCS *in vivo* control measurement of the eGFP-mRFP1 fusion protein (upper row) and eGFP and mRFP1 monomers (lower row) expressed in HeLa cells. (A), (B) and (C) the intensity image, slow and fast diffusion coefficient and relative concentration maps, respectively. (D) and (E) correlation functions and fits for the fusion protein and the monomers, horizontal dashed lines indicate the cross-correlation explained by cross-talk.

To verify the SPIM-FCCS method, we have performed *in vivo* control measurements of the eGFP and mRFP1 monomers and eGFP-mRFP1 fusion protein. Besides, these constructs were used to obtain reference minimum and maximum relative concentrations *p*_*AB*_ *in vivo*, respectively, similarly as we obtained *q* values for these constructs by confocal FCCS. Fig 3A–3C show a typical intensity image and maps of the fast and slow diffusion coefficient components and relative concentration for the negative and positive controls, i.e. monomers and fusion protein, respectively. Typical correlation functions and fits are shown in Fig 3D and 3E. The (mean±SD) of the fast diffusion coefficient D_fast_, obtained over 20 cell measurements, was (30±20) μm^2^/s for the eGFP-mRFP1 fusion protein and (38±15) μm^2^/s for the eGFP and mRFP1 monomers. The slow diffusion coefficient D_slow_ was (0.39±0.2) μm^2^/s for the fusion protein and (0.38±0.2) μm^2^/s for the monomers. The relative concentration *p*_*AB*_ was (0.29±0.15) for the fusion protein and served as a reference for complete dimerization *in vivo*. The separately expressed eGFP and mRFP1 gave a relative concentration *p*_*AB*_ of (0.08±0.08). This value was assumed as background due to spectral cross-talk between the channels and served as a reference for no interaction. The results for all 20 cells are presented in the supplementary information (S1 and S3 Figs). The results are comparable to the confocal FCCS measurements on the same construct [7, 43], which indicates that SPIM-FCCS is a viable method for studying protein dynamics in live cells.

**Fig 4:**
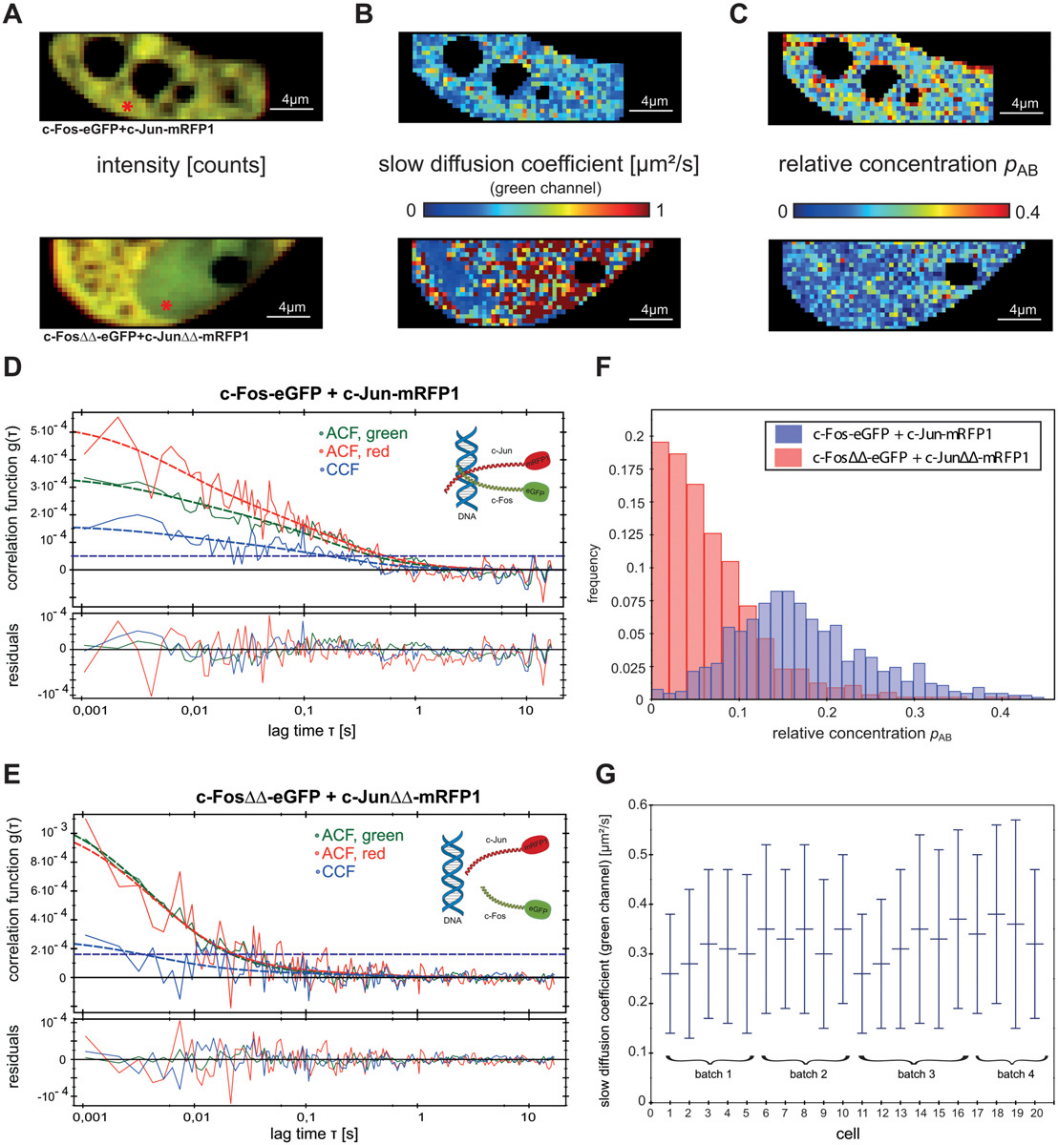
SPIM-FCCS *in vivo* measurements of AP-1 wild type proteins and deletion mutants in HeLa cell. (A) intensity images, (B) diffusion coefficients and (C) relative concentration maps for AP-1 wild type proteins (top) and its mutants (bottom), respectively. (D) and (E) auto- and cross-correlation functions and fits, obtained for locations indicated by the red mark in the intensity images. The horizontal dashed lines indicate the cross-correlation explained by cross-talk. (F) histogram of relative concentrations for the wild type and deletion mutant proteins shown in (C). (G) mean±SD of c-Fos-eGFP slow diffusion coefficient for all 20 cells.

SPIM-FCCS results of a typical HeLa cell with AP-1 wild type proteins c-Fos-eGFP and c-Jun-mRFP1 and one with deletion mutants are presented in Fig 4. The results for all 20 cells were similar and are shown in the supplementary information (S5 and S7 Figs). To illustrate a typical magnitude of noise, bleaching and background we included in supplementary information examples of intensity traces for the green and red channels for a representative pixel with high and a pixel with low interaction (S10 Fig).

Fig 4A shows the intensity images for the c-Fos-eGFP and c-Jun-mRFP1 (top) and the mutants (bottom). The upper image clearly indicates that c-Fos and c-Jun are localized only in the cell nucleus. This is due to the nuclear localization sequence (NLS) that is present in all full-length AP-1 proteins. The deletion mutants are distributed over the cytoplasm and the nucleus, as they are lacking the NLS. For the cells containing c-Fos-eGFP and c-Jun-mRFP1, the globally fitted diffusion coefficient was (0.28±0.1) μm²/s in the green and (0.3±0.1) μm²/s in the red channel for the slow and (18±10) μm²/s in the green and (23±6) μm²/s in the red channel for the fast component (Table 2). The diffusion maps (Fig 4B) show the slow diffusive component represented formally by a diffusion coefficient. This should be taken as a phenomenological indication of mobility, since the actual random motion of proteins in the crowded intracellular environment is obviously more complex than can be described by one simple diffusion process. For the c-Fos-eGFP and c-Jun-mRFP1 mutants there is a lot of noise on the slow diffusion coefficient because of its much lower amplitude compared to the wild type. The diffusion coefficients of the slow component for all 20 c-Fos-eGFP / c-Jun-mRFP1 cells are shown in Fig 4G. As previously described [43], we identified the slow diffusion coefficient with AP-1 proteins bound to immobile structures in the cell nucleus, most probably DNA. However, we also detected two diffusing components in the mutants with diffusion coefficients (0.38±0.2) μm²/s in the green and (0.4±0.2) μm²/s in the red channel for the slow, and (22±10) μm²/s in the green and (23±7) μm²/s in the red channel for the fast component. The slow component might therefore partially correspond to non-specifically trapped proteins. The relative concentration *p*_*AB*_, averaged over 20 cells, was (0.21±0.18) for the c-Fos-eGFP and c-Jun-mRFP1, and (0.13±0.07) for the deletion mutants.

The well-structured map of relative concentrations for the cell expressing c-Fos-eGFP and c-Jun-mRFP1 (Fig 4C top) clearly indicates stronger interactions, while the map of the cell expressing deletion mutants (Fig 4C bottom) is indicative of almost no interactions: this could be expected since their dimerization and DNA-binding domains were deleted. The small amount of interaction, i.e. low values of *p*_*AB*_ are the consequence of cross-correlation due to cross-talk (horizontal lines marked in the correlation function graphs). Fig 4D and 4E show auto- and cross-correlation functions obtained for locations indicated by the red mark in the intensity images. The two histograms in Fig 4F illustrate the difference in the relative concentration *p*_*AB*_ between the c-Fos-eGFP and c-Jun-mRFP1, and its mutants. As already shown by the *p*_*AB*_ maps, the mutants exhibited no significant interaction.

The slow and fast component diffusion coefficients, relative concentrations *p*_*AB*_ and relative cross-correlation amplitudes *q*, obtained by SPIM-FCCS and confocal FCCS, for the c-Fos-eGFP and c-Jun-mRFP1, their mutants, eGFP-mRFP1 fusion protein, and eGFP, mRFP1 monomers are summarized in Table 2. The values are averaged over 20 HeLa cell measurements each.

**Fig 5:**
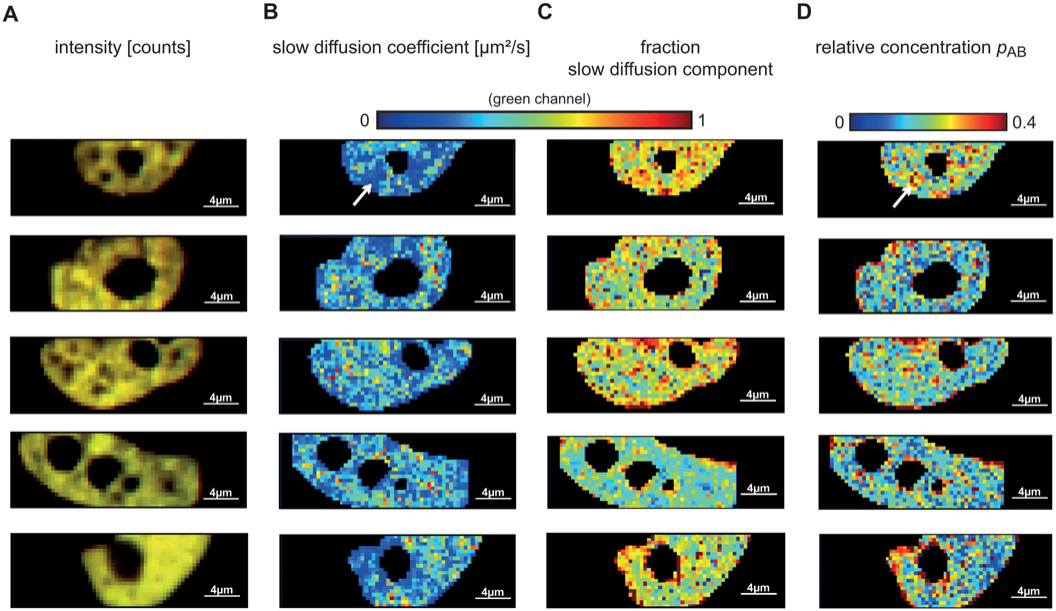
Typical SPIM-FCCS *in vivo* measurements of the AP-1 wildtype proteins c-Fos-eGFP and c-Jun-mRFP1 in five cells. (A) intensity images, (B) slow component diffusion coefficient maps showing the diffusion coefficients across the cells, (C) fraction of the slow diffusion component and (D) relative concentration maps.

We tried to infer a specific pattern of dimerization sites within a cell transfected with c-Fos-eGFP and c-Jun-mRFP1. Fig 5 shows intensity images (Fig 5A) and maps of the slow diffusion coefficient (Fig 5B), fraction of the slow diffusion component (Fig 5C) and the relative concentration *p*_*AB*_ (Fig 5D) for five different cells. The maps reveal areas in the nucleus where c-Fos-eGFP and c-Jun-mRFP1 exhibit higher interaction and a lower diffusion coefficient (one such area is highlighted by an arrow in Fig 5B and 5D), as well as areas of higher relative concentration and fraction of the slow diffusion component. Most of these areas are around the nuclear envelope and around the nucleolus. This finding may lead to the conclusion that c-Fos and c-Jun are associated with heterochromatin, which is located at the periphery of the nucleus and around the nucleolus. However, from the maps of the 20 analyzed cells, no other characteristic pattern of higher interaction areas could be established.

**Fig 6:**
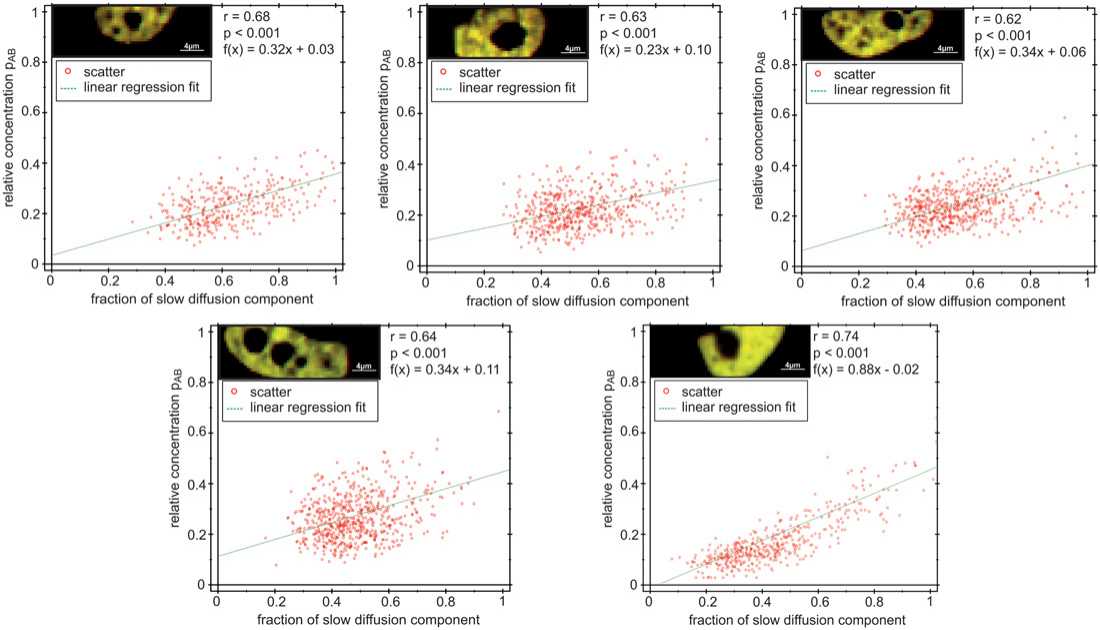
Scatter plots illustrating the relationships between relative concentrations and fraction of the slow diffusing component for the five cells shown in Fig 5.

Visual inspection of the diffusion maps, like the ones in Fig 5, suggested a correlation between the relative concentration *p*_*AB*_—indicating dimer formation—and the overall diffusion time, which appeared slower in the regions where strong dimerization occurred. To assess this effect quantitatively, we fixed both slow and fast diffusion coefficients to their average values across all pixels from the previous fit and fitted the data by varying only one parameter, the fraction of the slowly diffusing component. We then plotted the *p*_*AB*_ against the fraction of the slow component and fitted linear regression lines to the scatter plots for all 20 cells. Fig 6 shows scatter plots for the five cells in Fig 5, while scatter plots for all 20 cells are presented in S9 Fig.

For 20 cell measurements, the Pearson correlation coefficient *r* ranged from r = 0.54 to r = 0.75. The significance of the correlation was checked with the p-value against the null hypothesis of no correlation, which for c-Fos-eGFP and c-Jun-mRFP1 was always *p*<0.001. Thus the relative concentration and the fraction of the slow component are significantly correlated, strongly supporting the view that dimerization is a prerequisite for Fos/Jun binding.

**Fig 7:**
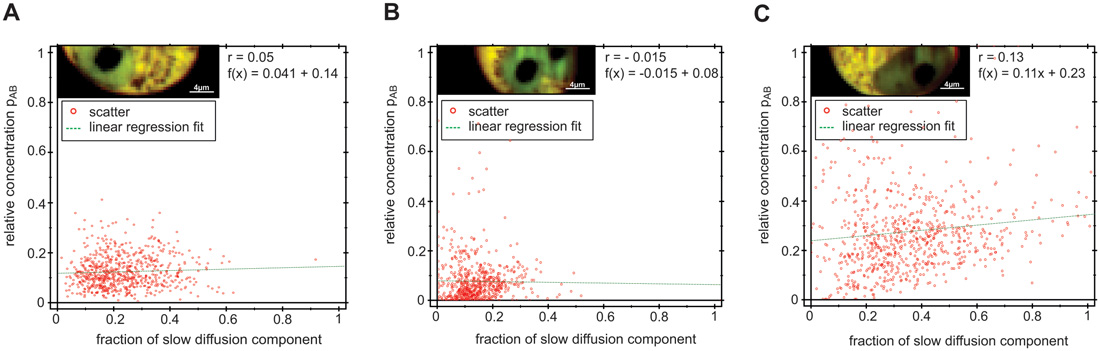
Scatter plots illustrating the relationships between relative concentration and fraction of the slow diffusing component. (A) c-FosΔdimΔDNA-eGFP and c-JunΔdimΔDNA-mRFP1, (B) eGFP and mRFP1 monomers and (C) eGFP-mRFP1 fusion protein expressed in HeLa cell.

As a control, we analysed the data from the c-Fos-eGFP and c-Jun-mRFP1 deletion mutants, eGFP and mRFP1 monomers and the eGFP-mRFP1 fusion protein. These constructs may either exhibit no interaction at all (deletion mutants and eGFP/mRFP1 monomers) or 100% interaction (fusion protein). Therefore, the relative concentration should not be correlated with the diffusion behavior. Fig 7 presents the fraction of the slow diffusing component and relative concentrations in the form of scatter plots with a superimposed linear regression fit. The Pearson correlation coefficients were very low, indicating no significant correlation between the two parameters. Similar results were obtained for all measured cells and are presented in the supplementary information (S2, S4, S6 and S9 Figs).

## Conclusion

Dual-color fluorescence cross-correlation spectroscopy on an in-house constructed single plane illumination microscope (SPIM-FCCS) allowed us for the first time to measure the mobility and interaction of two proteins *in vivo* and simultaneously across an entire cell nucleus. The method provides rather detailed maps of diffusion coefficients and relative concentrations, indicating regions of slow and fast diffusion, and strong and weak interaction, within the live cell.

Here we used the SPIM-FCCS system to study the motion and interaction of the transcription factors c-Fos and c-Jun, which participate in the regulation of several cellular processes, including differentiation, proliferation, apoptosis and oncogenesis [33–40]. Fos forms heterodimers with Jun-related proteins, whereas c-Jun forms homodimers and heterodimers with all Jun and Fos related proteins [52–59]. Whether Fos can also form homodimers is still an unresolved issue; studies of Fos homodimerization are currently underway in our laboratory.

Previous *in vivo* studies of the association of AP-1 transcription factors and their binding to DNA used single point confocal FCCS [43, 44]. They showed that c-Fos and c-Jun interact in living HeLa cells, using two autofluorescent protein tags. The disadvantage of confocal FCCS is that only a single measurement can be taken at a time, whereas to establish the spatially varying dynamics and interactions of proteins, experiments must be performed at many spots in parallel within the entire cell. SPIM-FCCS allows such measurements.

As a two-dimensional detector we used a fast, high quantum efficiency EMCCD camera. Auto- and cross-correlation curves were obtained by fitting a global 2-component model, which yielded a fast and a slow diffusion coefficient for each pixel. We obtained the relative concentration *p*_*AB*_ and presented the data as spatially resolved maps of c-Fos and c-Jun mobility and interaction. The diffusion coefficients for c-Fos-eGFP and c-Jun-mRFP1 were (18±10) μm²/s in the green and (23±6) μm²/s in the red channel for the fast and (0.32±0.15) μm²/s in the green and (0.3±0.1) μm²/s in the red channel for the slow diffusing component, which are comparable to the diffusing component measured by confocal FCCS [43]. The slow diffusing component is associated with dimerization of transcription factors and their binding to DNA.

To validate the results on c-Fos and c-Jun proteins, we performed several controls. Confocal FCCS measurements at several spots of HeLa cells expressing either c-Fos-eGFP and c-Jun-mRFP1 or the mutants c-FosΔΔ-eGFP and c-JunΔΔ-mRFP1 resulted in correlation functions very similar to those obtained by SPIM-FCCS. Second, HeLa cells transfected with control vectors pSV-eGFP-mRFP1 (a fusion protein of the two dyes separated by a 7-AA linker), and pIRES2-eGFP-mRFP1 (expressing the dyes separately), gave the limits for the cross-correlation amplitudes in the case of 100% and 0% interaction. Finally, we performed SPIM-FCCS measurements with the mutants c-FosΔΔ-eGFP and c-JunΔΔ-mRFP1. In these mutants, where the dimerization and DNA-binding domains were deleted, we found no interaction and no DNA binding.

We found a significant spatial correlation in the SPIM-FCCS images between the fraction of the slow diffusion component (= DNA binding) and relative concentration *p*_*AB*_ (= dimerization), allowing us to map the diffusion properties and localization of c-Fos and c-Jun interactions and the regions of interactions between AP-1 transcription factors and DNA.

As discussed in [32, 33], the SPIM-FCCS technique expands the repertoire of imaging tools with measurements of the mobility and interaction of molecules in live cells. Besides that, the method might find use not only in cell biological studies, but could also be valuable for high-throughput detection of protein-drug interactions in live cells, since it would allow detection of the association and dissociation of target proteins [60] and as functional *in vivo* screening for inhibitor or enhancers of biomolecular interactions [61].

## Supporting Information

S1 Fig SPIM-FCCS *in vivo* measurements of eGFP and mRFP1 monomer protein in HeLa cells: intensity images, relative concentration maps and fast and slow diffusion coefficients maps for all 20 measured cells. (PDF)

S2 Fig Scatter plots illustrating the relationships between the fraction of the slow diffusing component, where D1 and D2 were fixed at average for the fit, and relative concentration *p*_*AB*_ of eGFP and mRFP1 monomer protein in HeLa cells for all 20 measured cells. Each graph shows a scatter plot where a point indicates values obtained at one pixel, and the linear regression fit.
(EPS)

S3 Fig SPIM-FCCS *in vivo* measurements of eGFP-mRFP1 fusion protein in HeLa cells: intensity images, relative concentration maps and fast and slow diffusion coefficients maps for all 20 measured cells. (PDF)

S4 Fig Scatter plots illustrating the relationships between the fraction of the slow diffusing component, where D1 and D2 were fixed at average for the fit, and relative concentration *p*_*AB*_ of eGFP-mRFP1 fusion protein in HeLa cells for all 20 measured cells. Each graph shows a scatter plot where a point indicates values obtained at one pixel, and the linear regression fit.
(EPS)

S5 Fig SPIM-FCCS *in vivo* measurements of AP-1 deletion mutants c-FosΔdimΔDNA-eGFP and c-JunΔdimΔDNA-mRFP1 in HeLa cells: intensity images, relative concentration maps and fast and slow diffusion coefficients maps in the green channel for all 20 measured cells. (PDF)

S6 Fig Scatter plots illustrating the relationships between the fraction of the slow diffusing component, where D1 and D2 were fixed at average for the fit, and relative concentration *p*_*AB*_ of AP-1 deletion mutants c-FosΔdimΔDNA-eGFP and c-JunΔdimΔDNA-mRFP1 in HeLa cells for all 20 measured cells. Each graph shows a scatter plot where a point indicates values obtained at one pixel, and the linear regression fit.
(EPS)

S7 Fig SPIM-FCCS *in vivo* measurements of AP-1 wildtype proteins c-Fos-eGFP and c-Jun-mRFP1 in HeLa cells: intensity images, relative concentration maps and fast and slow diffusion coefficients maps in the green channel for all 20 measured cells. (PDF)

S8 Fig SPIM-FCCS *in vivo* measurements of AP-1 wildtype proteins c-Fos-eGFP and c-Jun-mRFP1 in HeLa cells: intensity images, relative concentration maps and fraction of the slow diffusion component maps for all 20 measured cells. (PDF)

S9 Fig Scatter plots illustrating the relationships between the fraction of the slow diffusing component, where D1 and D2 were fixed at average for the fit, and relative concentration *p*_*AB*_ of AP-1 wildtype proteins c-Fos-eGFP and c-Jun-mRFP1 in HeLa cells for all 20 measured cells. Each graph shows a scatter plot where a point indicates values obtained at one pixel, and the linear regression fit.
(EPS)

S10 Fig Examples of intensity traces before (B, E) and after (C, F) bleaching correction for representative pixels (indicated by arrow) with high relative concentration (top) and low relative concentration (bottom) for the green and the red channels. (EPS)
