# Supplementary material for: Imaging Fos-Jun Transcription Factor Mobility and Interaction in Live Cells by Single Plane Illumination-Fluorescence Cross Correlation Spectroscopy
Source: PLoS One. 2015 Apr 14;10(4):e0123070. doi: 10.1371/journal.pone.0123070 (PMC4397054; doi:10.1371/journal.pone.0123070)

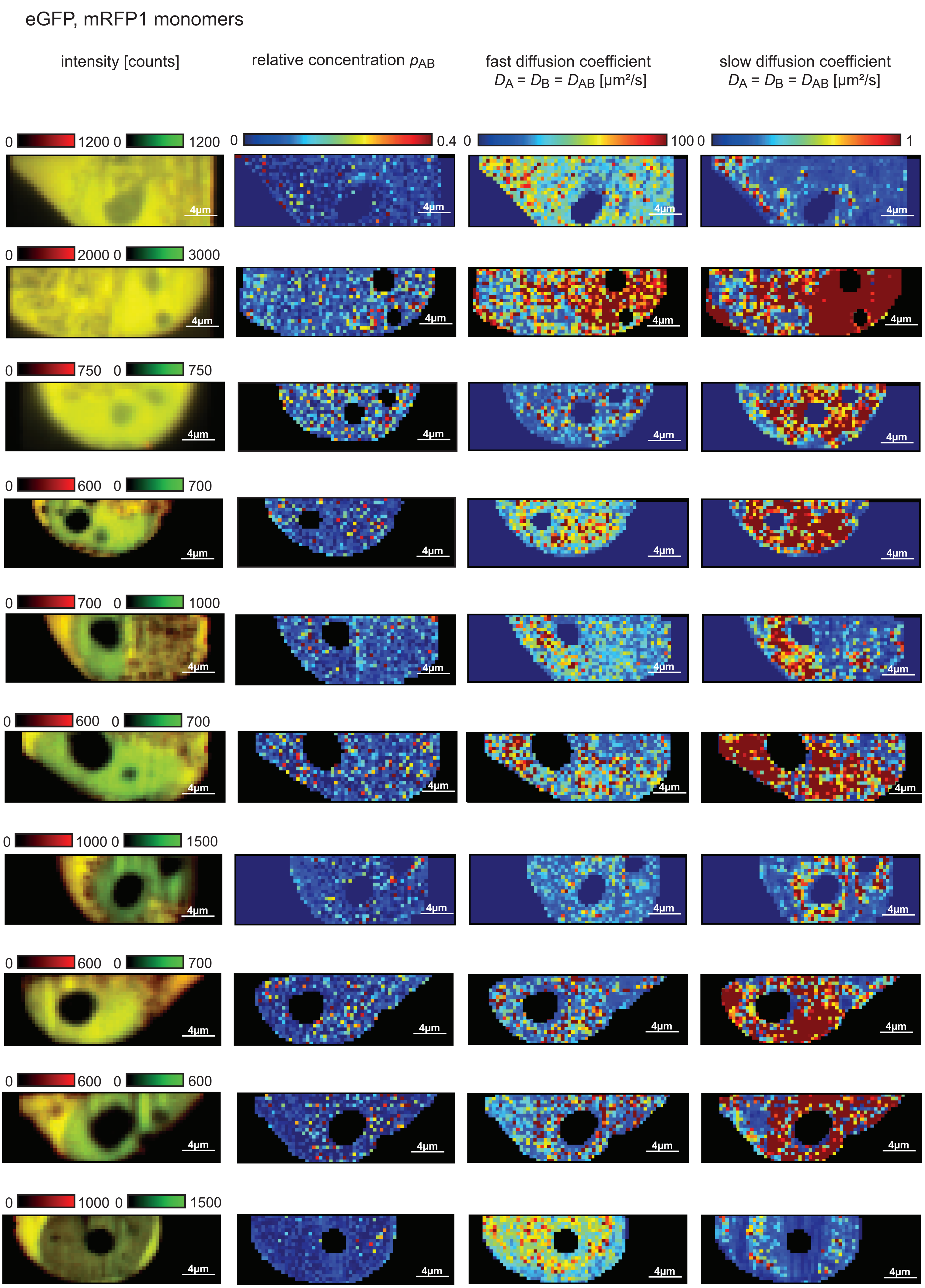

eGFP, mRFP1 monomers
intensity [counts]
relative concentration p_{AB}
fast diffusion coefficient
$D_A = D_B = D_{AB}$ [µm²/s]
slow diffusion coefficient
$D_A = D_B = D_{AB}$ [µm²/s]
0 1200 0 1200
0 0.4
0 100
0 1
0 2000 0 3000
0 750 0 750
0 600 0 700
0 700 0 1000
0 600 0 700
0 1000 0 1500
0 600 0 700
0 600 0 600
0 1000 0 1500
4µm

eGFP, mRFP1 monomers

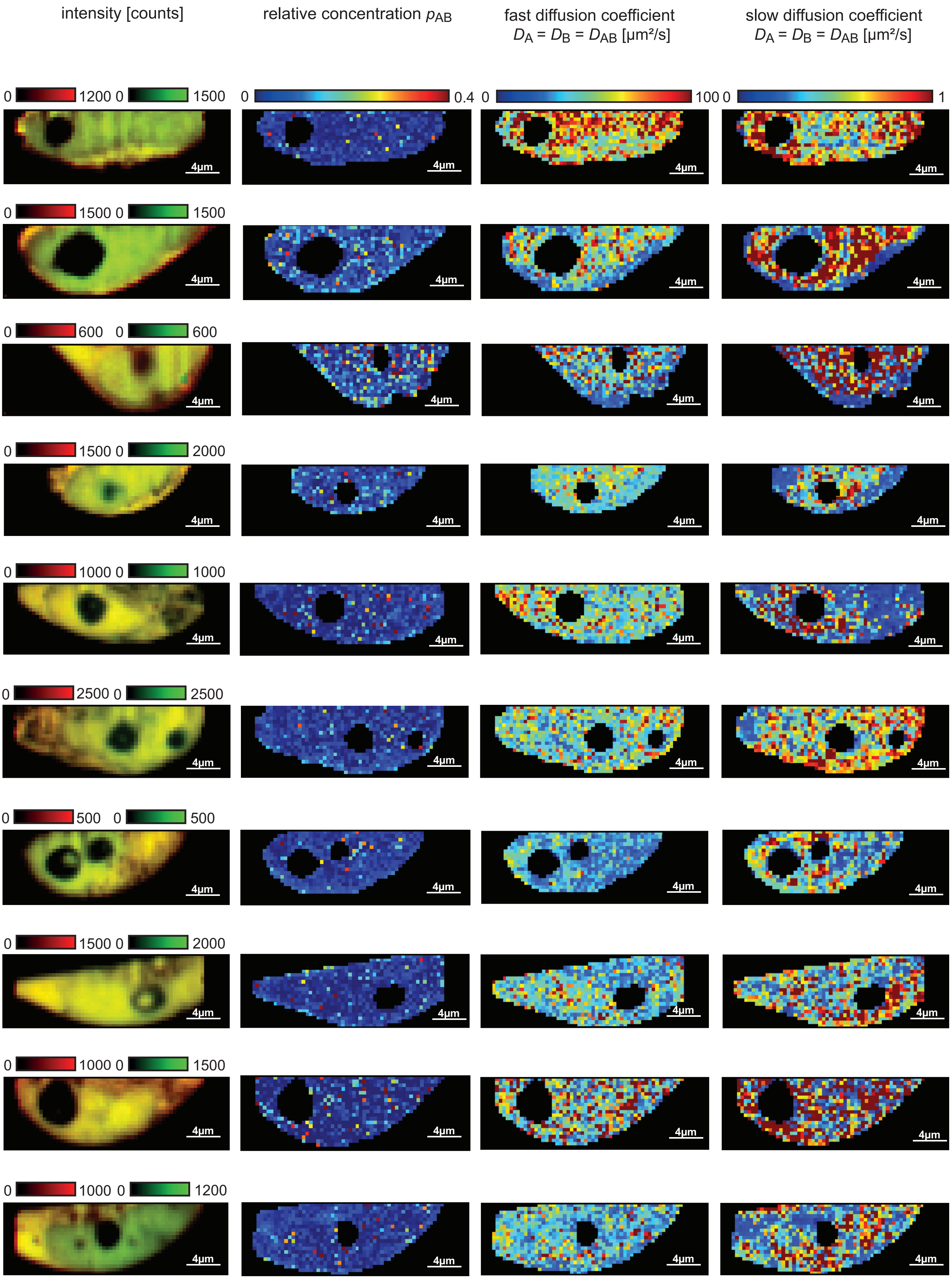

Supplement: S1 Fig — (PDF) [file pone.0123070.s001.pdf]
