# Supplementary figures and images for: Imaging Fos-Jun Transcription Factor Mobility and Interaction in Live Cells by Single Plane Illumination-Fluorescence Cross Correlation Spectroscopy
Source: PLoS One. 2015 Apr 14;10(4):e0123070. doi: 10.1371/journal.pone.0123070 (PMC4397054; doi:10.1371/journal.pone.0123070)

# eGFP-mRFP1 fusion protein

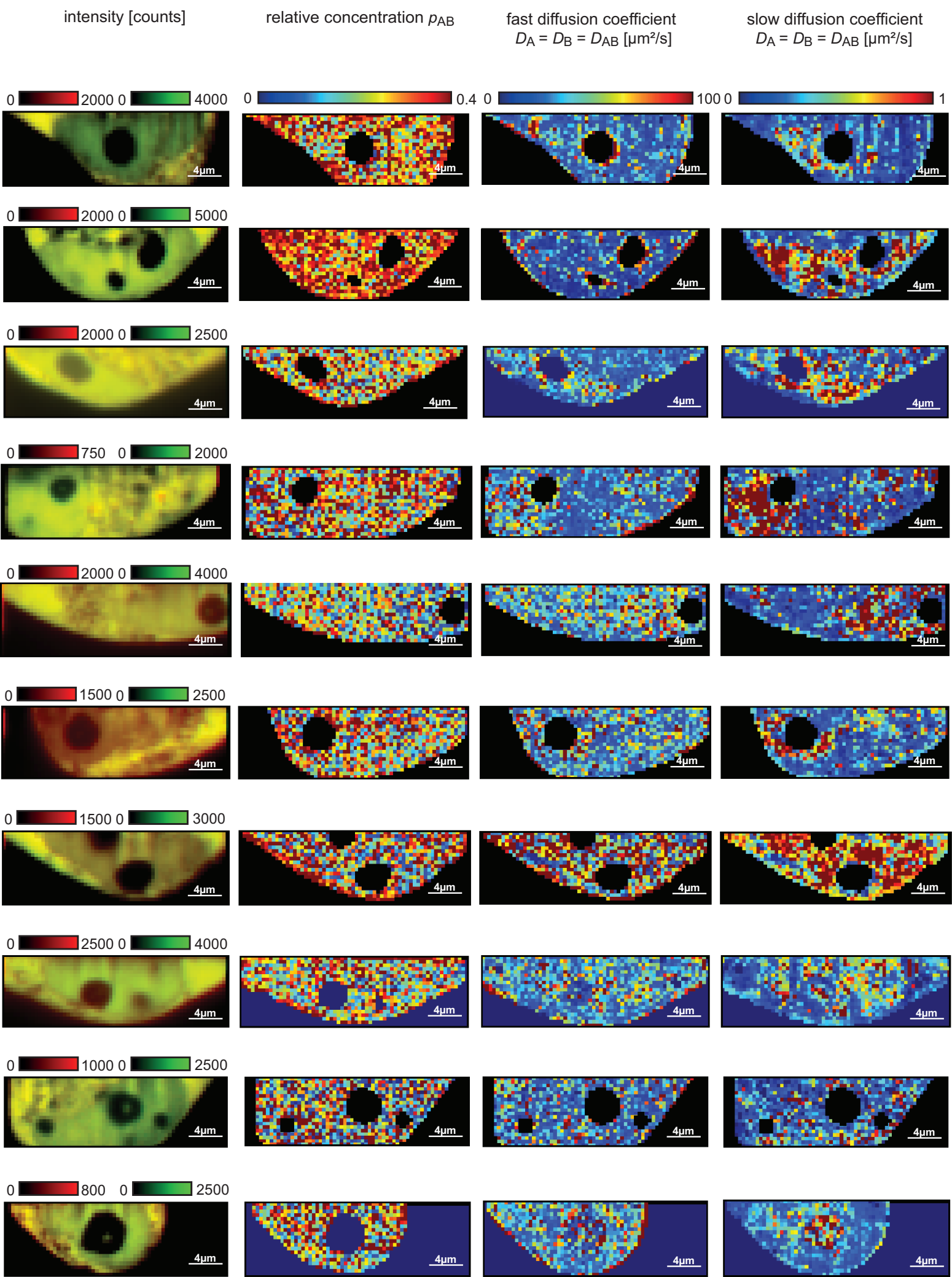

# eGFP-mRFP1 fusion protein

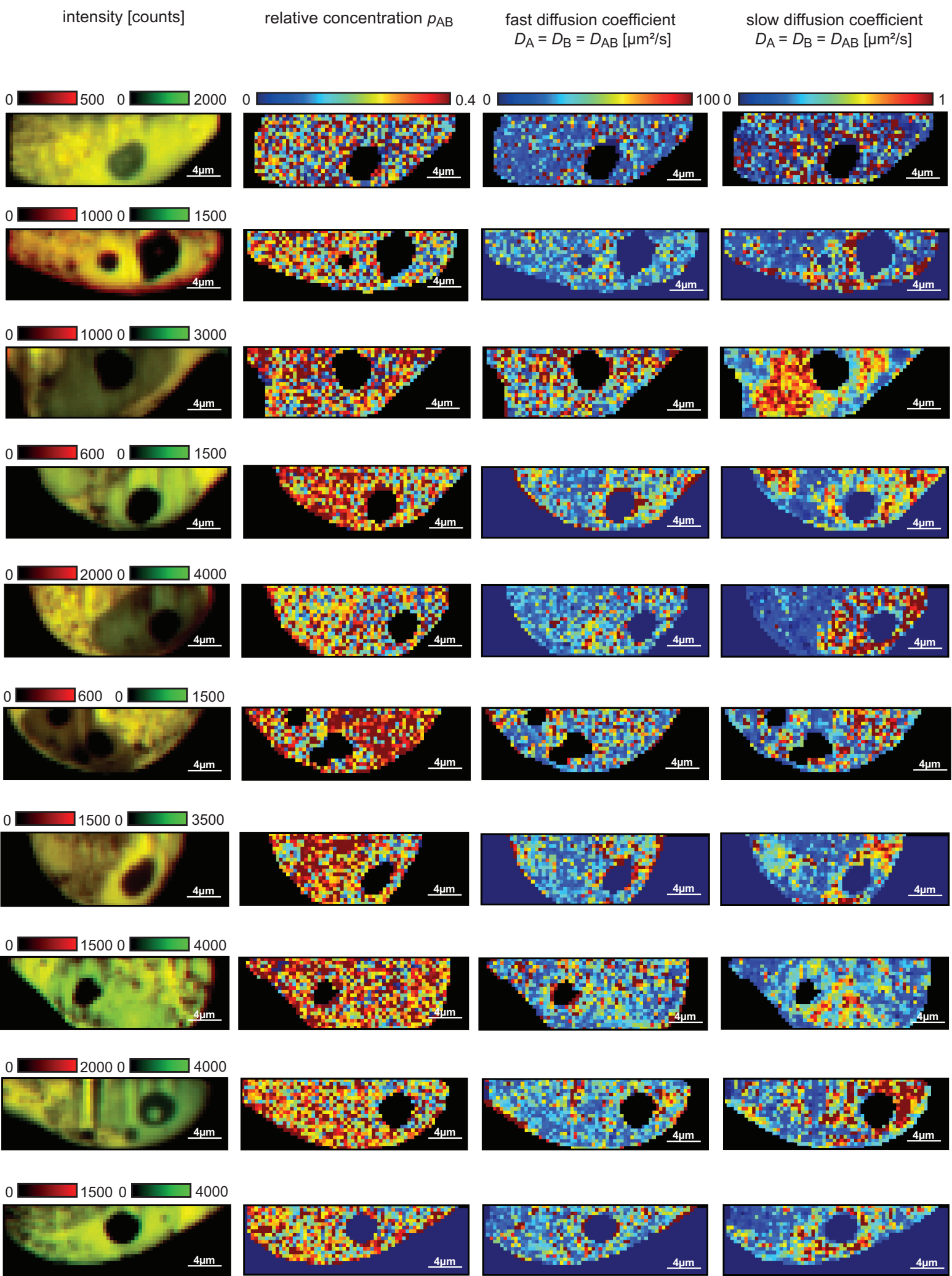

Supplement: S3 Fig — (PDF) [file pone.0123070.s003.pdf]

# c-Fos $\Delta\Delta$ -eGFP+c-Jun $\Delta\Delta$ -mRFP1

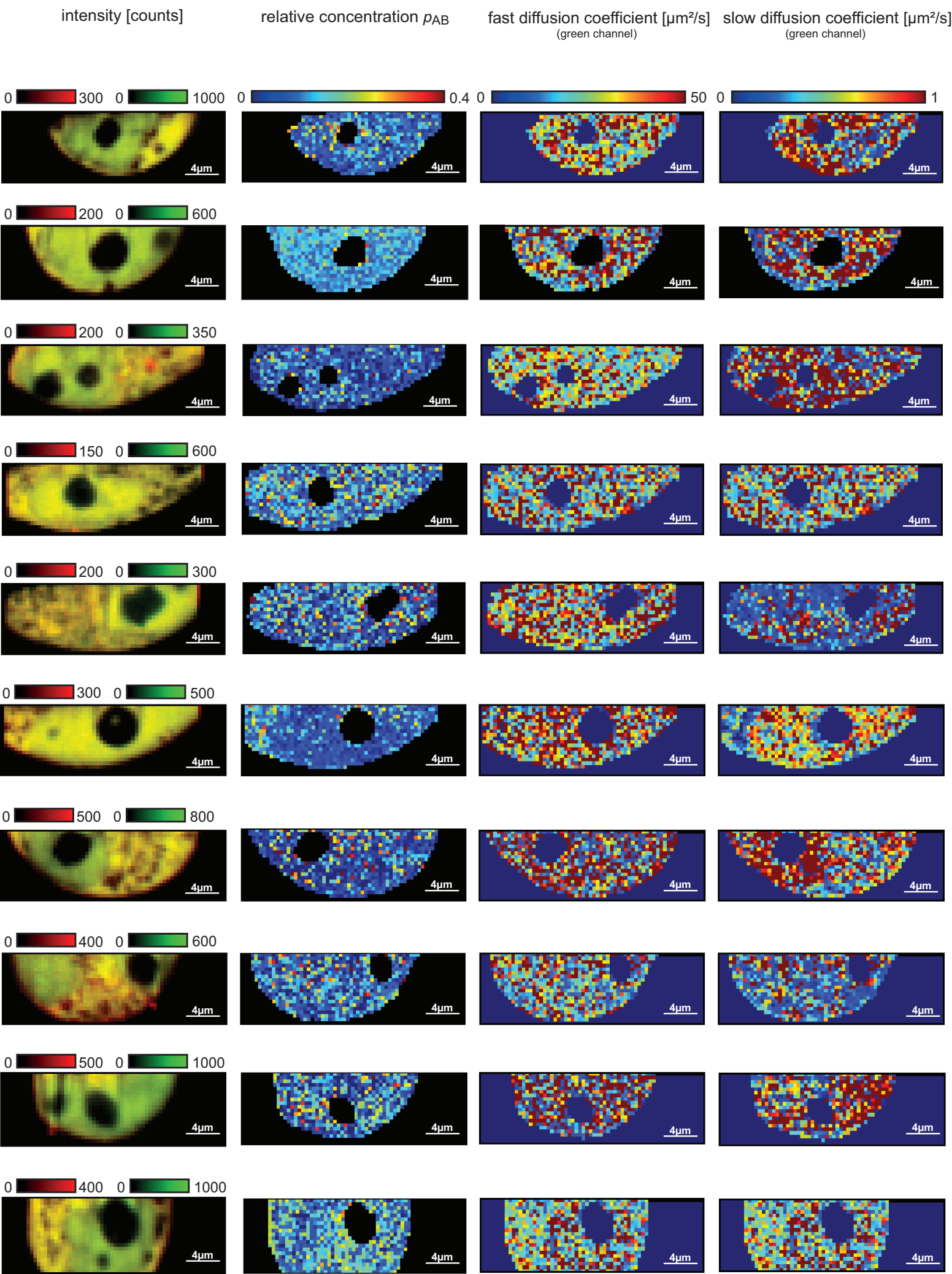

# c-Fos $\Delta\Delta$ -eGFP+c-Jun $\Delta\Delta$ -mRFP1

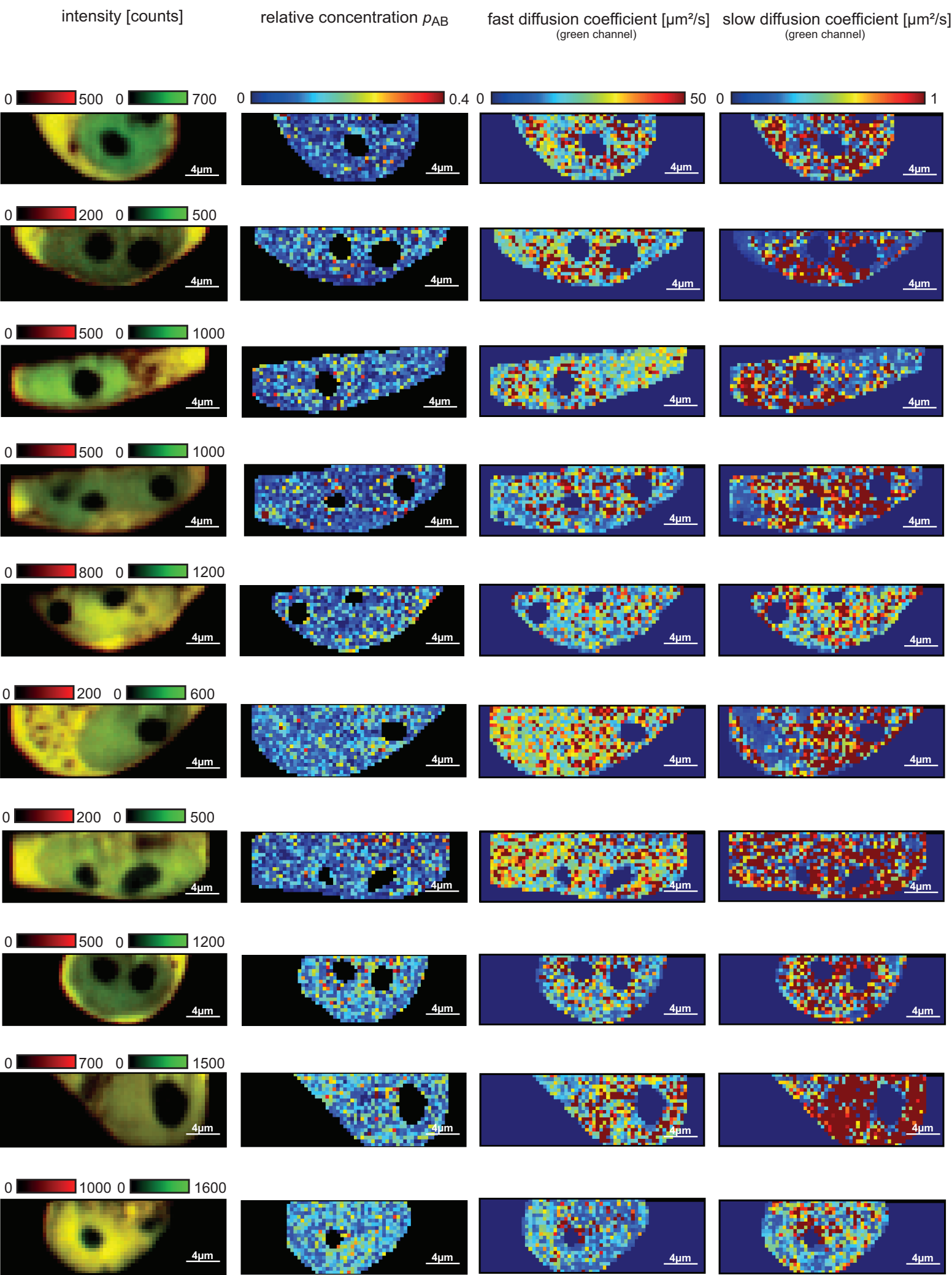

Supplement: S5 Fig — (PDF) [file pone.0123070.s005.pdf]

# c-Fos-eGFP+c-Jun-mRFP1

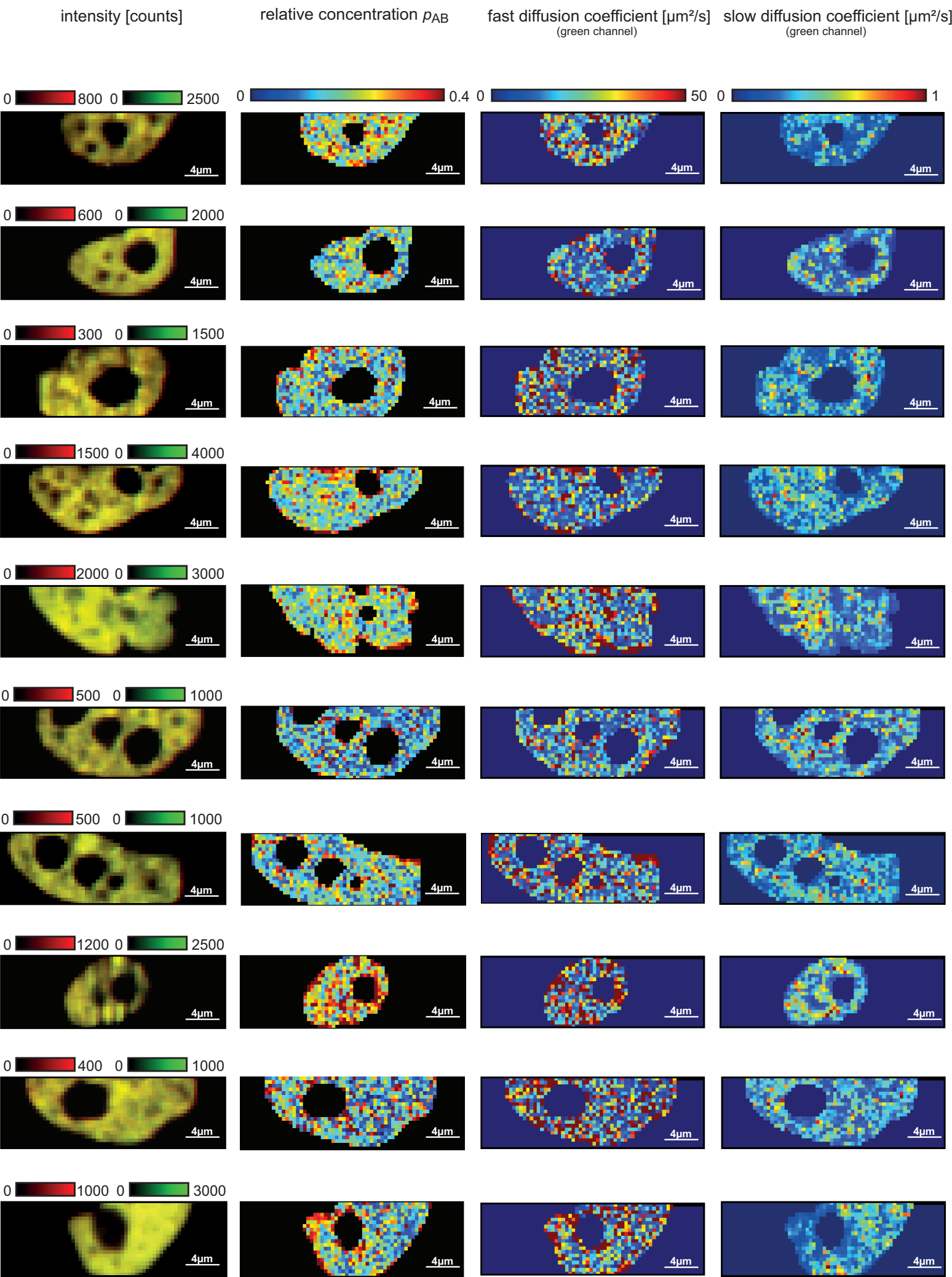

# c-Fos-eGFP+c-Jun-mRFP1

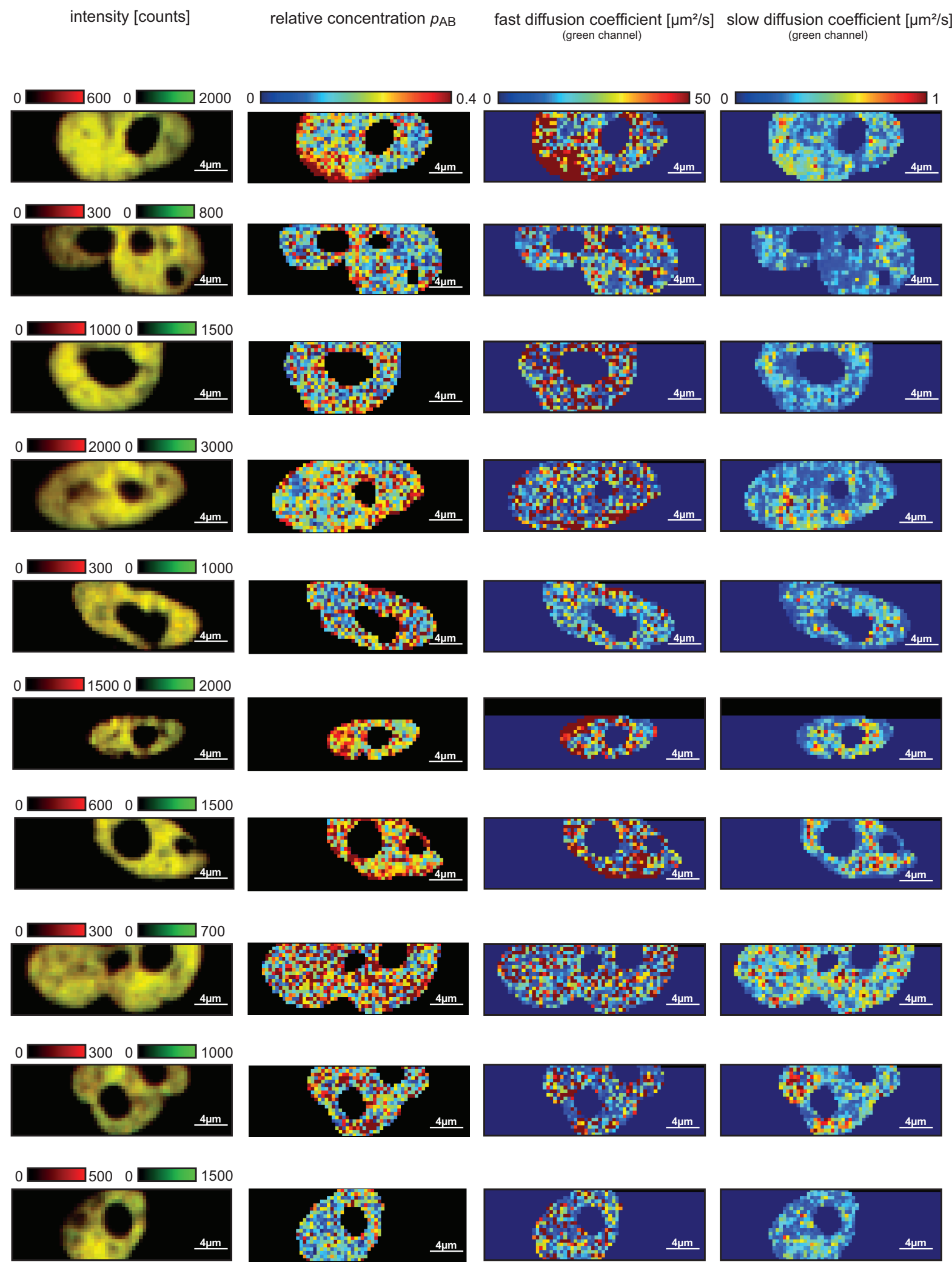

Supplement: S7 Fig — (PDF) [file pone.0123070.s007.pdf]

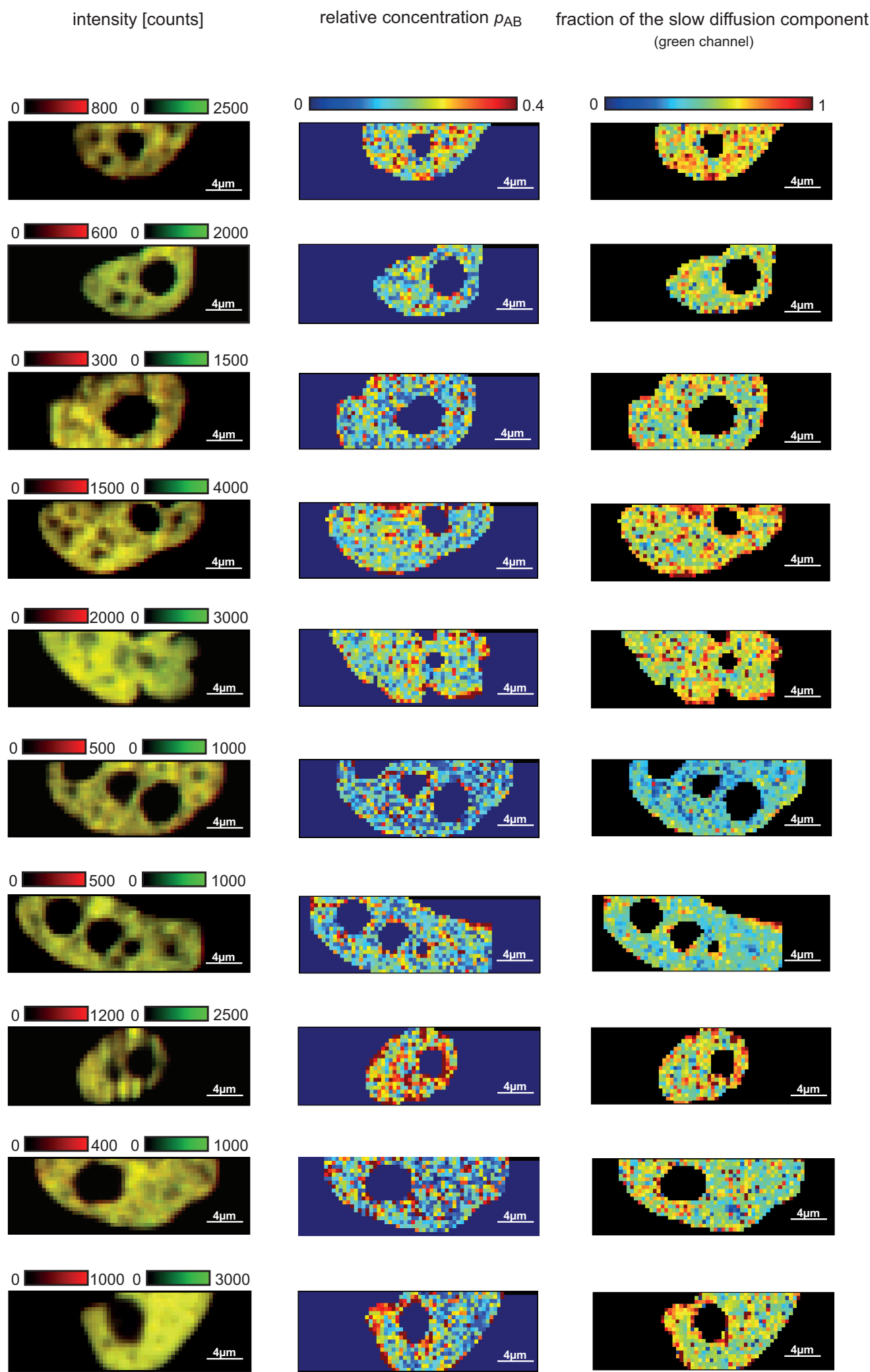

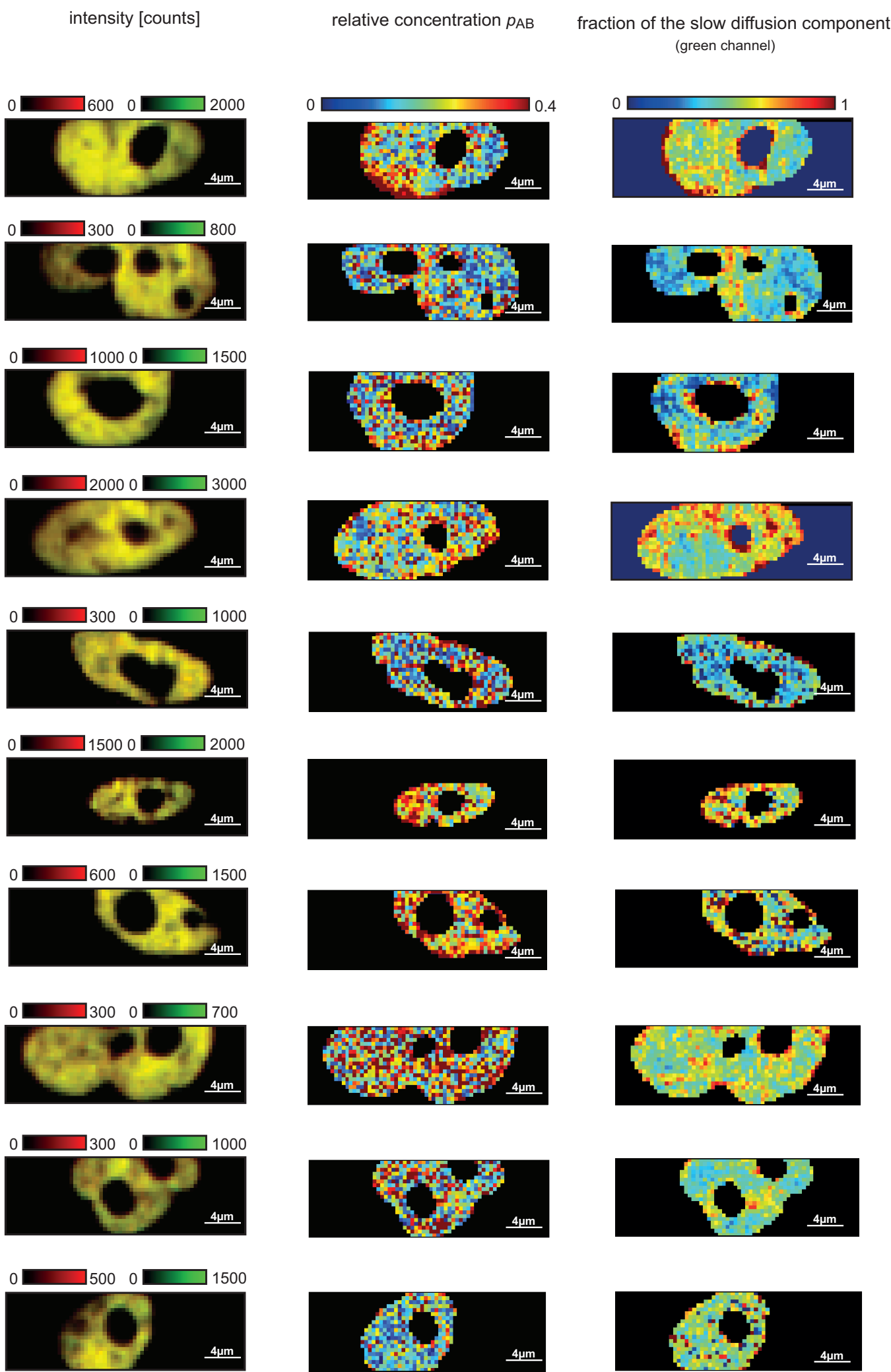

Supplement: S8 Fig — (PDF) [file pone.0123070.s008.pdf]
